# Occurrence of the millipede genus *Tonkinosoma* Jeekel, 1953 in China, with the description of the first presumed troglobitic species of this genus (Diplopoda, Polydesmida, Paradoxosomatidae)

**DOI:** 10.3897/zookeys.742.23471

**Published:** 2018-03-12

**Authors:** Weixin Liu, Sergei Golovatch

**Affiliations:** 1 Department of Entomology, College of Agriculture, South China Agricultural University, 483 Wushanlu, Guangzhou 510642, China; 2 Institute for Problems of Ecology and Evolution, Russian Academy of Sciences, Leninsky pr. 33, Moscow 119071, Russia

**Keywords:** Guangxi, Guizhou, key, new record, new species, *Tonkinosoma*, troglobite

## Abstract

The genus *Tonkinosoma* Jeekel, 1953 has hitherto been known to contain only two species, both from northern Vietnam. *T. flexipes* Jeekel, 1953, the type species of the genus, is recorded from Guangxi, southern China, for the first time. *T. tiani* **sp. n.**, a presumed troglobite, is described from caves in Guizhou, southwestern China. A key is presented to all three species of the genus.

## Introduction

The millipede genus *Tonkinosoma* Jeekel, 1953 was originally proposed to encompass only a single species, *T. flexipes* Jeekel, 1953, from northern Vietnam (Jeekel 1953). The tribe Tonkinosomatini Jeekel, 1968 was erected to comprise not only *Tonkinosoma*, but also a few other genera (Jeekel 1968). However, this tribe has since been merged first with Sulciferini Attems, 1898 (cf. Golovatch 2014) and, later, with Chamberliniini Wang, 1956 (cf. Golovatch 2015a). Nguyen (2011), when reviewing Tonkinosomatini in the scope of the fauna of Vietnam, described a second species from northern Vietnam: *T. jeekeli* Nguyen, 2011.

At present, *Tonkinosoma* can be diagnosed as a genus of the Himalayan and southeast Asian tribe Chamberliniini characterized by the gonopods which show a postfemoral part demarcated basally by an indistinct (*T. flexipes*) or distinct (*T. jeekeli*) geniculation cingulum and distally at least by a lateral sulcus. Unlike the other contribal genera, the femorite is long and slender and, like the postfemoral part, it is devoid of outgrowths, the seminal groove runs all along the mesal face of the femorite, the solenomere is long and flagelliform, and the solenophore is a long, hyaline, folded lobe that shows a lamina medialis and a lamina lateralis, both sheathing the solenomere. As in most Chamberliniini, both solenomere and solenophore are usually subcircular (Chen et al. 2010; Nguyen and Korsós 2011; Golovatch 2014).

Golovatch (2014) questioned the attribution of *T. jeekeli* to *Tonkinosoma*, but now we rather believe that he somewhat misinterpreted the gonopodal structure of *T. flexipes* as described and illustrated by Jeekel (1953). Instead we follow Nguyen (2011) and consider both *T. flexipes* and *T. jeekeli* to represent congeners.

Prompted by the discovery of both *T. flexipes* and a third, new species of *Tonkinosoma* in southern China, the latter species the first to be found in caves, their descriptions are provided below. We also provide a key to all three presently known species of this genus.

## Material and methods

All specimens used in this study were collected in southern China and preserved in 95% ethanol. Most of the type and non-type material is deposited in the Zoological Collection of the South China Agricultural University, Guangzhou, China (**SCAU**), with several samples also donated to the Zoological Museum, Moscow State University, Moscow, Russia (**ZMUM**), as indicated below.

Observation and dissections were performed using a Leica S8 APO stereo microscope. The line drawings were prepared with a Leica MZ125 microscope and a camera lucida attached to the microscope.

Photographs were taken with a Keyence VHX-5000 digital microscope, and further edited using Adobe Photoshop CS5.

## Taxonomy

### Key to species of *Tonkinosoma*

**Table:** 

1	Body with a distinct colour pattern, yellowish brown to reddish brown (Figs 1–2). Epigean species	**2**
–	Body uniformly yellowish to pallid (Fig. 4). Presumably troglobitic species	***T. tiani* sp. n.**
2	A large, median, subquadrate process between ♂ coxae 4 and two small, independent tuberculations between ♂ coxae 5 (Fig. 2F)	***T. flexipes***
–	Only two small and independent processes between ♂ coxae 4, without any modifications between ♂ coxae 5	***T. jeekeli***

#### Tonkinosoma flexipes

Taxon classificationAnimaliaPolydesmidaParadoxosomatidae

Jeekel, 1953

Figs 1 , 2 , 3

Tonkinosoma flexipes Jeekel, 1953: 1, figs 1–4.
Tonkinosoma flexipes – Nguyen 2011: 68.

##### Material examined.

9 ♂, 1 ♀ (SCAU), 2 ♂ (ZMUM), China, Guangxi, Hechi City, Fengshan County, Jinya Town, Hangdong Village, 24°37'44"N, 106°51'26"E, 500 m a.s.l., 14.VI.2014, leg. Mingyi Tian, Weixin Liu, Haomin Yin & Xiaozhu Luo.

##### Remarks.

**Figure 1.**
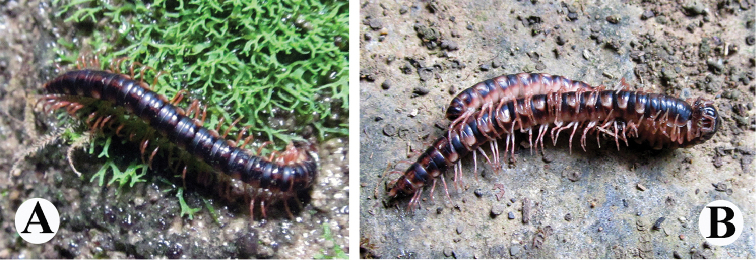
*Tonkinosoma flexipes* Jeekel, 1953 in nature. **A** a ♂ **B** a mating pair.

**Figure 2.**
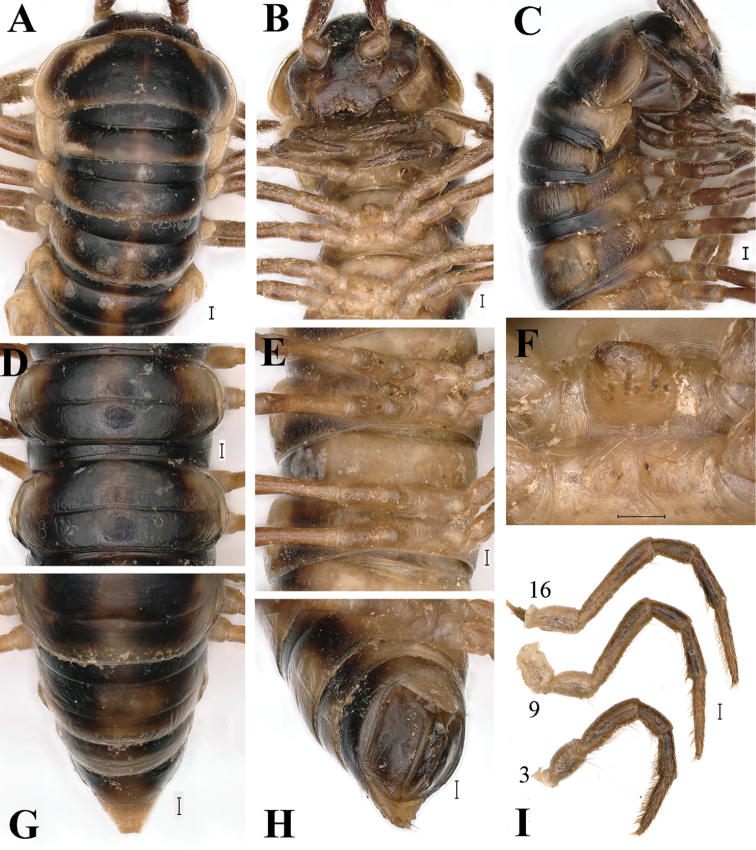
*Tonkinosoma flexipes* Jeekel, 1953, ♂. **A–C** anterior part of body, dorsal, ventral and lateral views, respectively **D, E** midbody segments; dorsal and subventral views, respectively **F** sternite V, ventral view **G, H** posterior part of body, dorsal and ventrolateral views, respectively **I** legs 3, 9, 16, anterior views. Scale bar: 0.2 mm.

**Figure 3.**
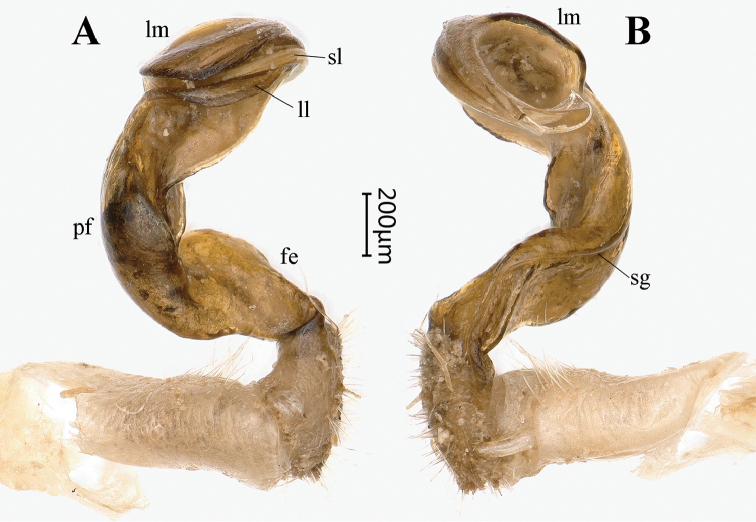
*Tonkinosoma flexipes* Jeekel, 1953, ♂. **A, B** right gonopod, lateral and mesal views, respectively. Abbreviations: fe = femorite; ll = lamella lateralis; lm = lamella medialis; pf = postfemur; sg = seminal groove; sl = solenomere.

This is the type species of *Tonkinosoma* hitherto known only from a highly detailed original description, based on the male holotype and two paratypes, one male and one female, all from Mt Manson, Langson Province, northern Vietnam (Jeekel 1953). Above is only the second record of *T. flexipes*, a species new to the fauna of China, but this is hardly too surprising because it comes from a place quite close to the border with northern Vietnam. The new samples almost fully agree with the original description (Jeekel 1953), but our material is remarkably smaller (19–28 mm *vs.* 37–47 mm). The habitus and gonopod structure (Figs 1–3) are illustrated to document the species’ identity. Among the main diagnostic characters of *T. flexipes*, the following seem to be especially noteworthy to complement the only available description: integument strongly shining; metazonae with several longitudinal striae above paraterga (Fig. 2C); pleurosternal carinae present on segments 2–4 in both sexes (Fig. 2C); a large, median, subquadrate process between ♂ coxae 4 and two small, independent tuberculations between ♂ coxae 5 (Fig. 2F); legs ca 1.6 (♂) or 1.2 (♀) times as long as midbody height, tarsal brushes present on all ♂ legs (Fig. 2I); gonopodal postfemoral part only indistinctly demarcated, lamina lateralis well-developed only in the proximal part of the solenophore (Fig. 3).

#### Tonkinosoma tiani sp. n.

Taxon classificationAnimaliaPolydesmidaParadoxosomatidae

http://zoobank.org/87580449-1285-4C19-B810-8169522704D8

**Figure 4.**
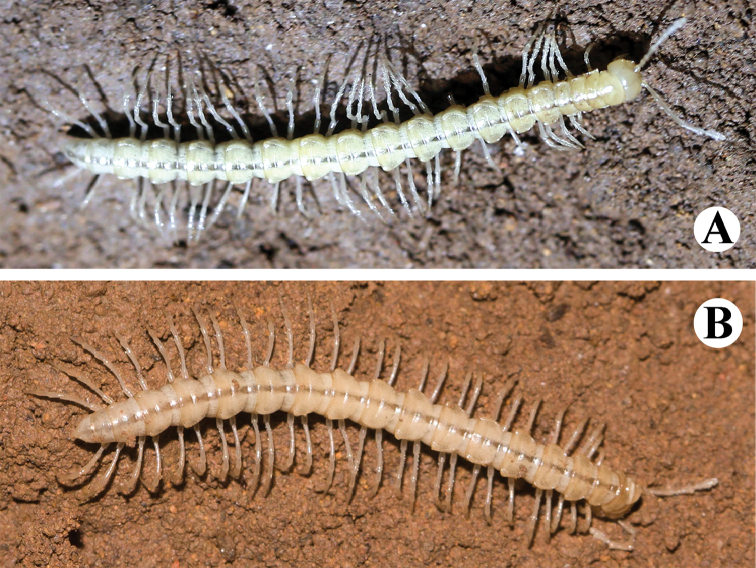
*Tonkinosoma tiani* sp. n. in nature **A, B** two different ♂ paratypes.

**Figure 5.**
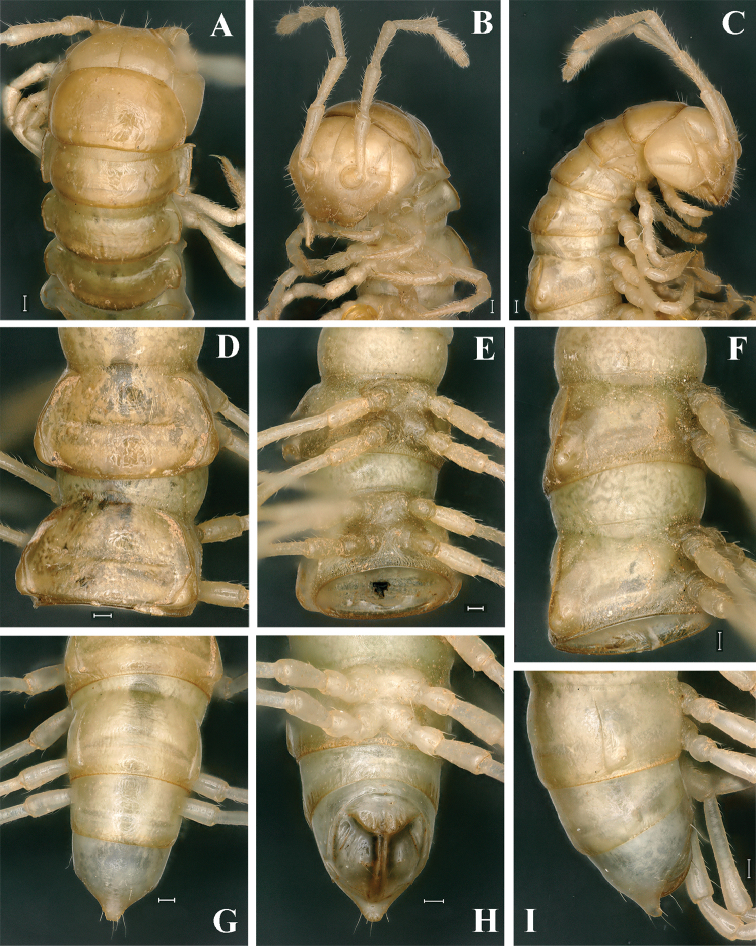
*Tonkinosoma tiani* sp. n., ♂ paratype from Ganhan Cave. **A–C** anterior part of body, dorsal, ventral and lateral views, respectively **D–F** midbody segments; dorsal, ventral and lateral views, respectively **G–I** posterior part of body, dorsal, ventral and lateral views, respectively. Scale bar: 0.2 mm.

**Figure 6.**
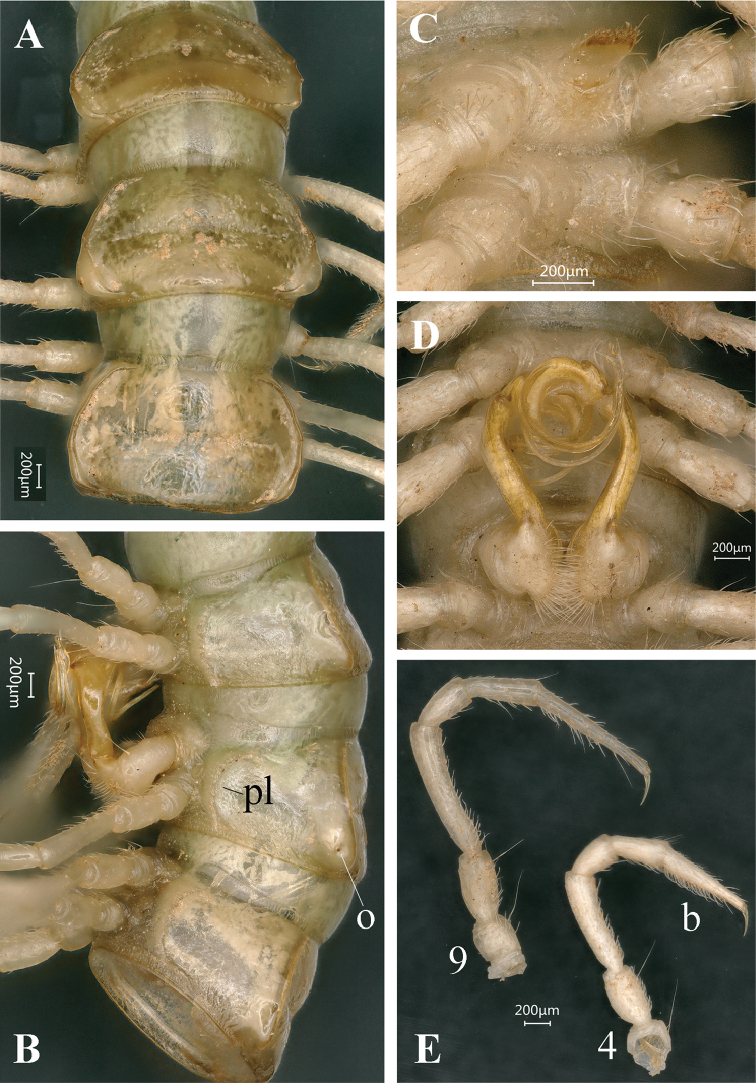
*Tonkinosoma tiani* sp. n., ♂ paratype from Ganhan Cave. **A, B** segments 6–8, dorsal and lateral views, respectively **C** sternite V, ventral view **D** gonopods *in situ*, ventral view **E** legs 4 and 9, anterior view. Abbreviations: b = tarsal brush; o = ozopore; pl = pleurosternal carina.

**Figure 7.**
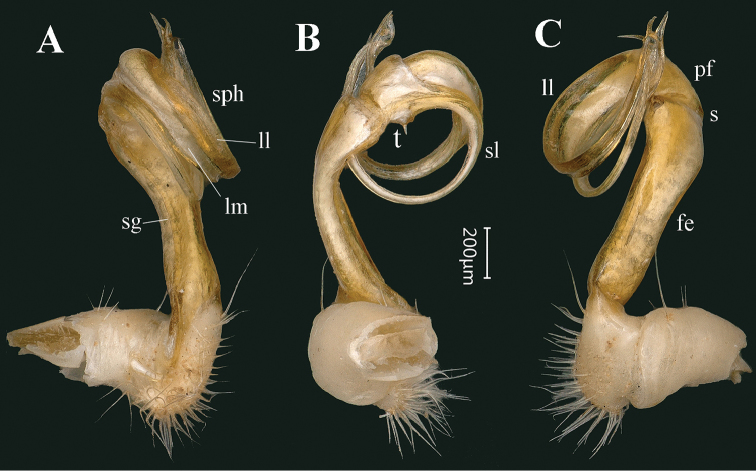
*Tonkinosoma tiani* sp. n., ♂ paratype from Ganhan Cave. **A–C** left gonopod, mesal, anterior and lateral views, respectively. Abbreviations: fe = femorite; ll = lamella lateralis; lm = lamella medialis; pf = postfemur; s = sulcus; sg = seminal groove; sl = solenomere; sph = solenophore; t = tooth.

**Figure 8.**
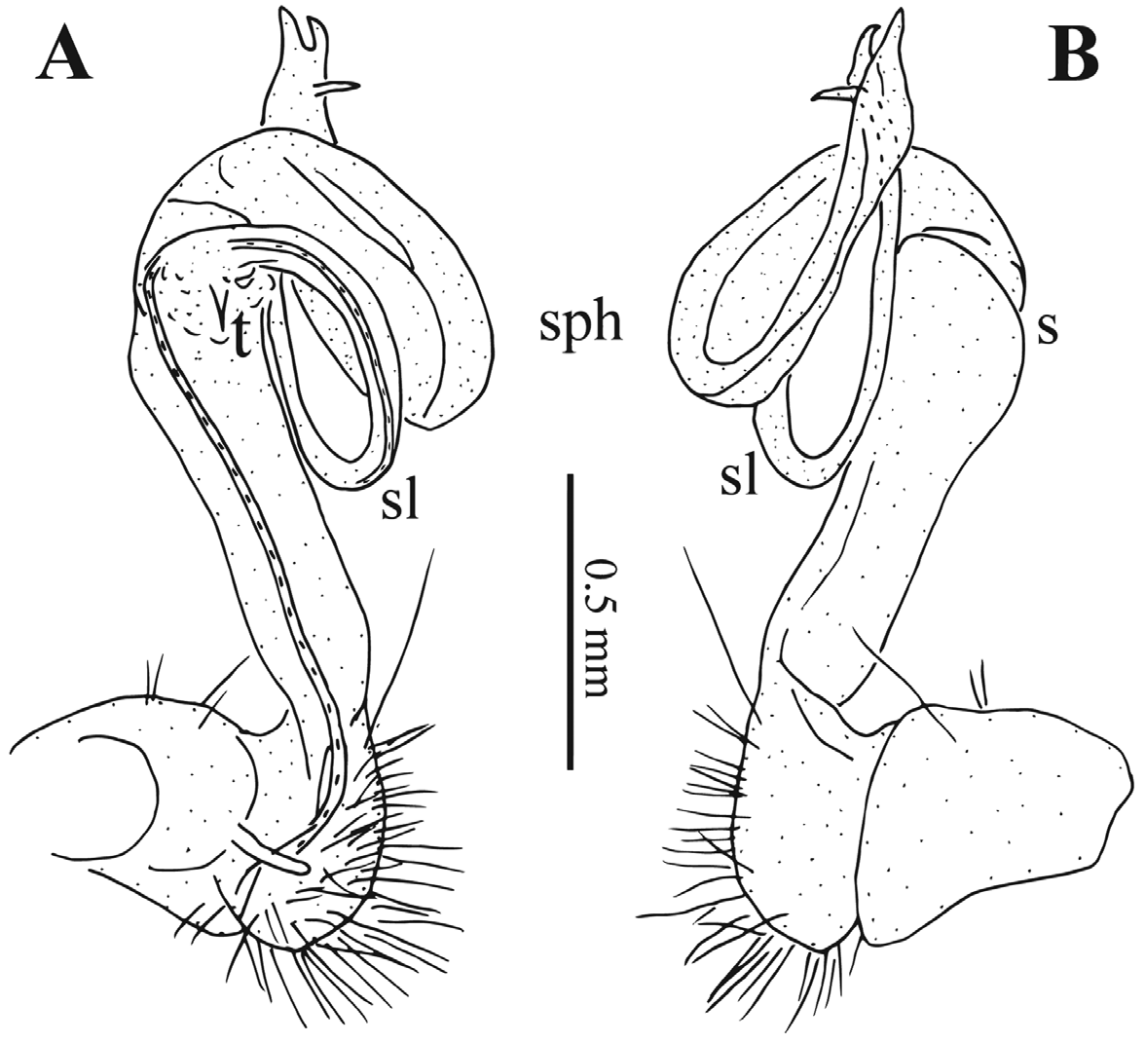
*Tonkinosoma tiani* sp. n., ♂ paratype from Ganhan Cave. **A, B** left gonopod, submesal and lateral views, respectively. Abbreviations: s = sulcus; sl = solenomere; sph = solenophore; t = tooth.

Figs 4 , 5 , 6 , 7 , 8

##### Type material.

Holotype ♂ (SCAU), China, Guizhou Province, Qianxinan, Anlong County, Sayu Town, Ganhan Dong Cave, 25°11'25"N, 105°19'31"E, 1250 m a.s.l., 12.V.2017, leg. Mingyi Tian, Weixin Liu, Xiaozhu Luo, Pingjing Yang & Yanyi Pu.

##### Paratypes.

9 ♂, 20 ♀ (SCAU), same data as holotype. 8 ♂ (SCAU), same county, Longguang Town, Fengyan Dong Cave, 25°10'05"N, 105°13'50"E, 1400 m a.s.l., 12.V.2017; 13 ♂, 4 ♀ (SCAU), 1 ♂, 1 ♀ (ZMUM), Guizhou, Xingyi City, Wushan Town, Xiaozi Dong Cave, N 25°06'43", E 104°46'32", 1750 m a.s.l., 14.V.2017; all leg. Mingyi Tian, Weixin Liu, Xiaozhu Luo, Pingjing Yang & Yanyi Pu.

##### Name.

To honour Prof. Mingyi Tian, one of the collectors from South China Agricultural University.

##### Diagnosis.

This new species differs from its congeners in showing a largely unpigmented body. It seems to be especially similar to *T. jeekeli* on account of the particularly elongate and subcircular solenophore and solenomere, but differs by the strongly developed pleurosternal carinae present until segment 17 in both sexes, by an evident, subtrapeziform process between ♂ coxae 4, and the gonopod with a small and sharp tooth near the base of the solenomere.

##### Description.

Lengths of both sexes ca 25–27 mm, widths 1.6–1.8 and 2.0–2.2 mm (♂) or 1.8–2.0 and 2.2–2.5 mm (♀) on pro- and metazonae, respectively. Holotype ca 27 mm long, and 1.8 and 2.2 mm wide on midbody pro- and metazonae, respectively.

*Live coloration* rather uniformly yellowish to pale (Fig. 4).

*Body* with 20 segments. In width, collum < head = segment 3 < 2 = 4 < 5–7 < 8–16, thereafter body increasingly tapered towards telson.

*Head*: frons densely pilose, vertex smooth, epicranial suture distinct (Fig. 5A–B). Antennae long and slender, reaching behind body segment 4 when extended posteriorly; in length, antennomere 2 > 3 > 5 > 4 > 6 > 1 > 7 (Fig. 5B–C).

*Collum* with 4+4 short setae at anterior margin. Following metaterga with traces of at least 1+1 setae before transverse sulcus, but pattern mostly vague and setae abraded. Paraterga of collum small, but evident, rounded. Paraterga 2 well-developed, directed down, with 4–5 clear lateral incisions on each side, frontolateral corner much sharper (Fig. 5A). Paraterga 3–6 each with three small, lateral incisions (Figs 5A), following paratergal incisions indistinct (Figs 5D, G, 6A). Calluses of paraterga 5–18 very thin in poreless segments, slightly thicker and sinuate in dorsal view in caudal 1/3 (ozopore position) of pore-bearing ones (Figs 5D, F, 6A–B); paraterga 19 nearly suppressed, but its ozopores clear (Fig. 5I).

*Integument* shining, texture of prozonae finely micro-alveolate. Stricture between pro- and metazonae broad and shallow, clearly ribbed (Figs 5D, 6A–B).

*Pore* formula normal (5, 7, 9, 10, 12, 13, 15–19), ozopores distinct, entirely lateral, lying inside an ovoid groove near caudal paratergal corner (Figs 5F, 6B).

*Transverse sulcus* incomplete on metaterga 4–7, more evident, complete and reaching bases of paraterga on metaterga 8–18 (Figs 5D, G, 6A). Axial line missing.

*Epiproct* tip truncated, with four spinnerets (Figs 5G–I). Paraproct with two setigerous knobs. Hypoproct roundly subtrapeziform, caudal setae distinctly separated, borne on evident knobs (Fig. 5H).

*Pleurosternal carinae* very strongly developed, present on segments 2–17 both in ♂ and ♀ (Figs 5C, F, 6B).

*Sterna* modestly setose, cross-impressions shallow (Fig. 5E). An evident, apically setose, subtrapeziform process between ♂ coxae 4 (Fig. 6C).

*Legs* long and slender, ca 2.5 (♂) or 2.0 (♀) times as long as midbody height. Tarsal brushes present only on ♂ legs 1–7, following legs normal, unmodified (Fig. 6D).

*Gonopods* (Figs 6D, 7, 8) simple. Coxite relatively short, about half as long as telopodite, poorly setose both distodorsally and distoventrally. Prefemoral portion densely setose as usual, about as long as coxite. Femorite (**fe**) long and slender, slightly curved mesally and faintly enlarged distally. An obvious demarcation sulcus (**s**) both laterally and dorsally between **fe** and a postfemoral portion (**pf**). Solenophore (**sph**) clearly coiled, circular, both lamina medialis (**lm**) and lamina lateralis (**ll**) well-developed and nearly entirely sheathing a similarly long and free solenomere (**sl**). Seminal groove (**sg**) running entirely on mesal side of femorite before moving onto **sl**, with a very small, sharp, mesal tooth (**t**) on **pf** near **sl** base.

##### Remarks.

The karstic Ganhan Dong cave where the holotype was taken is about 300 m long. All material was collected in areas of complete darkness.

Based on the largely unpigmented integument, the long legs (2.5 (♂) or 2.0 (♀) *vs.* 1.6 (♂) or 1.2 (♀) times as long as midbody height in *T. flexipes*) and the cave habitat, this species seems to be a troglobite.

## Discussion

The above record of *T. tiani* sp. n. in caves in southern China is remarkable at least in two ways. Firstly, the huge family Paradoxosomatidae only rarely occurs in caves, with only few presumably troglobitic species. The only exceptions are in the large genus *Desmoxytes* Chamberlin, 1923, which is very common both in epigean and subterranean environments across southeast Asia and China, and in the small genus *Piccola* Attems, 1953, with a few epigean species in Vietnam and Laos, and a single troglobitic one from Guangxi, China (Liu and Tian 2013; Golovatch 2015b). Secondly, biogeographically the situation concerning the distribution pattern of *Tonkinosoma* strongly resembles that not only of *Piccola*, but of still another millipede genus, i.e., *Pacidesmus* Golovatch, 1991 (Polydesmida, Polydesmidae). The latter genus has one high-mountain species in northern Thailand and a further eight, all presumed troglobites, in southern China (Golovatch and Geoffroy 2014).

Further research on cave millipedes of China will definitely reveal not only new interesting taxa, but more cases of remarkable distribution patterns.

## Supplementary Material

XML Treatment for Tonkinosoma flexipes

XML Treatment for Tonkinosoma tiani
